# Akzeptanz von Distance Working bei Führungskräften

**DOI:** 10.1365/s40702-021-00729-9

**Published:** 2021-04-16

**Authors:** Florian Bergsleitner, David Rückel

**Affiliations:** 1 AIM Austrian Institute of Management GmbH, Thomas-A-Edison-Straße 2, 7000 Eisenstadt, Österreich; 2 Johannes Kepler Universität, Alpenberger Str. 69, Linz, 4040 Österreich

**Keywords:** Führung, Distance Working, Akzeptanz, Konzept, Covid-19, Leadership, Distance Working, Acceptance, Concept, Covid-19

## Abstract

Geänderte Anforderungen an die Arbeitsplätze durch die globale Covid-Krise bedingen Veränderungen des Führungsstils. Losgelöst von althergebrachten Kontrollinstanzen definieren moderne Führungskräfte ihren Stil als „Digital Leader“ wesentlich umfassender und sind essenzieller Enabler, um Distance Working in Unternehmen zu etablieren. Obwohl Ressentiments gegen die Schaffung von Arbeitsplätzen im Distance Working bestehen, nehmen Führungskräfte gleichermaßen die Verantwortung für ihre Mitarbeiter und die Unternehmen wahr. Entsprechend ist es Ziel dieses Beitrags, die Bedenken und Herausforderungen von Führungskräften in Bezug auf Digital Leadership und Distance Working zu erheben und mittels Design Science aus dem aktuellen „State of the Field“ wissenschaftlicher Literatur verbunden mit einer beispielhaften Umsetzung ein Konzept zur Steigerung der Akzeptanz von Distance Working zu erstellen. Das entstandene Artefakt wird durch Interviews mit zehn Experten evaluiert. Technische Lösungen werden als Grundbedingung angesehen, die Vertrauenskultur deutlich stärker gelebt als dies von vorangegangen Stilen bekannt war. Führungskräfte empfinden beim Digital Leadership keinen Kontrollverlust, sie bringen den MitarbeiterInnen Vertrauen entgegen und monitoren deren Leistungen. Die Kommunikation wird um die außerdienstliche, soziale Komponente erweitert, da Führungskräfte erkennen, dass Mitarbeiterführung damit vertrauensvoller gelingen kann. Um das Unternehmen weiterhin gut am Markt positionieren und zukunftsorientiert agieren zu können, wünschen sich Führungskräfte im Gesetzgeber einen starken Verbündeten, der den Rahmen so gestaltet, dass sowohl Schutz des Mitarbeiters wie auch individuelle Lösungen gewährleistet bleiben.

## Abstract (en)

Changed demands on jobs and workplaces due to the global Covid crisis require changes in management styles. In contrast to traditional subvisory authorities, modern executives define their style as “digital leaders” much more comprehensively and are thereby essential enabling factors in establishing distance working in companies. Although there is resentment against the creation of jobs in distance working, managers take responsibility for both their employees and companies in many areas. Accordingly, the aim of this article is to raise the concerns and challenges of executives in relation to digital leadership and distance working and, by means of design science from the current “state of the field” scientific literature, combined with an exemplary implementation, a concept to increase the acceptance of distance working to create. The artifact created is evaluated through interviews with ten experts. Technical solutions are seen as crucial basic condition, the culture of trust is lived much more strongly than was known from previous styles. With digital leadership, managers do not feel any loss of control as they trust employees and monitor performance. The communication is expanded to include the off-duty, social component, as managers recognize that leadership can be more trustworthy. In order to continue to position the company well in the market and to be able to act in a future-oriented manner, executives want a strong ally in the legislature who creates the framework in such a way that both the protection of the employee and individual solutions are guaranteed.

## Einleitung

Ausgehend von globalen Phänomenen stellt die Digitalisierung von Prozessen und Arbeitsplätzen in Unternehmen sowie veränderte Ansprüche von Fachkräften an den Arbeitsmarkt Unternehmen im Allgemeinen und Führungskräfte im Speziellen vor neue Herausforderungen (Klammer et al. 2017). Parker (2020) konkretisiert diese Herausforderungen mit den Auswirkungen von Covid-19 auf den Arbeitsplatz in Bezug auf Kostenstruktur und Effizienz und stellt weiterführend fest, dass die Covid-19 Pandemie wohl die größte Auswirkung auf einzelne Individuen, das Sozialgefüge und die Wirtschaft seit über 100 Jahren darstellt. Dies führt dazu, dass Covid-19 einen Übergang zum „Distance Working“^1^ eingeleitet hat. Eine notwendige dezentralisierte Mitarbeiterführung (Digital Leadership) wird in einem ersten Schritt durch die Akzeptanz von Distance Working bei Führungskräften ermöglicht. Erfolgreich umgesetzt kann sich dies sowohl in einer orts-, als auch einer zeitunabhängigen Flexibilität widerspiegeln, bei dem stärkeren Fokus auf ein stabiles Angestelltenverhältnis als auf projektbezogene Beauftragungen. Ziel ist eine maximierte Work-Life-Balance von Mitarbeitern, welche in weiterer Folge zu einer gesteigerten Arbeitsmotivation sowie einer erhöhten Identifikation mit dem Unternehmen führt, was idealtypisch wiederum zu einer Steigerung der Attraktivität des Unternehmens am bzw. für den Arbeitsmarkt einhergeht (Günther 2017).

Weiterführend zeigen sich Bedenken hinsichtlich potenzieller Gefahren der Dezentralisierung, wie beispielsweise Kontrollverlust, unzureichende Kommunikation oder mangelnde Leistungserbringung. Demgegenüber stehen identifizierbare Chancen erhöhter Eigenverantwortlichkeit durch flexiblere Arbeitszeiten, verkürzte Arbeitswege und einer daraus resultierenden Motivationssteigerung (Janiesch et al. 2017). Latniak (2017) sieht in der zunehmenden Digitalisierung einen Treiber in Unternehmen, virtuelle Teamarbeit mit dem Vorteil zu nutzen, weltweit gezielt Mitarbeiter einbinden und beschäftigen zu können. Er rechnet mit zunehmendem Einsatz virtueller Teams, speziell in strategisch wichtigen Funktionen für Innovationsprozesse, wie beispielsweise der Unternehmens-IT. Auch Kötting (2019) sieht „gewichtige Vorteile bei der Durchsetzung eines digitalen Arbeitsplatzes für Unternehmen“: Einsparungspotential hinsichtlich Immobilienkosten und Nebenkosten, Verringerung der Abwesenheit und umweltverbessernde Einflüsse durch geringeres Pendeln.

Hierfür notwendig sind ein Überdenken bzw. Umgestalten des Führungsstils (Kordsmeyer et al. 2019), technische Lösungen sowie kommunikative und gesetzliche „Spielregeln“, um sich langfristig als erfolgreiches Unternehmen am Arbeitsmarkt präsentieren zu können (Rotzinger 2017). Deutlich gefordert sind hier die Führungskräfte, deren Bedenken eine Änderung vom Präsenzarbeitsplatz hin zu partiell orts- und zeitunabhängigen Arbeitsplätzen blockieren können und deren Expertise hinsichtlich technischer und organisatorischer Unterstützung von übergeordneten Einheiten in die Etablierung eines Digital Leadership einfließen muss.

Ziel dieser Arbeit ist es, subjektiv empfundene Hindernisse von Führungskräften in Bezug auf Mitarbeiterführung bei partiell orts- und zeitunabhängigen Dienstverhältnissen zu erkennen, technische und organisatorische Lösungen anzubieten und die Verwendung dieser Lösungen sowie die veränderte Wahrnehmung von Führungskräften in Bezug auf ihre Rolle im neuen Umfeld des Digital Leadership zu analysieren. Darauf aufbauend wird ein Konzept zur Steigerung der Akzeptanz von Distance Working bei Führungskräften geschaffen und ausführlich evaluiert.

Der vorliegende Beitrag gliedert sich wie folgt: Basierend auf einer kompakten Darstellung des Stands der Forschung zu den relevanten Themengebieten und der Vorstellung von Forschungsdesign und -methode wird das initiale Design des Konzepts als Artefakt vorgestellt und argumentiert. Darauf aufbauend stellen die Autoren die Erkenntnisse aus der Befragung von Experten zum Zweck der Evaluierung des Artefakts und das daraus resultierende überarbeitete Konzept vor. Abschließend werden die Ergebnisse interpretiert.

## Grundlagen aus Literatur und Praxis

Die Managementaufgaben des 21. Jahrhunderts haben sich aufgrund zunehmender Digitalisierung gegenüber den Aufgaben früherer Betrachtungszeiträume maßgeblich gewandelt. Offenkundig können Anpassungen zu Herangehensweisen von Führungskräften dem Tempo technologischer Entwicklungen nicht Stand halten. Neuerungen rufen nicht nur Begeisterung hervor, sondern oft Sorgen und Bedenken (Sikora 2017). Der Stand der Forschung in der Literatur identifiziert im Wesentlichen drei Faktoren, welche die Bedenken der Führungskräfte im Wandel hin zum Digital Leadership subsumieren: Unzureichende Kommunikation, mangelnde Kontrolle der Leistung sowie die Befürchtung inkorrekt dokumentierter oder unvollständiger Arbeitszeiten. Die beiden letzteren Faktoren lassen sich unter dem Stichwort Vertrauen zusammenfassen (Jäckel 2020).

Moderne Führung setzt stärker als traditionelle Führungsstile auf Vorbildfunktion und das Vorleben der gewünschten Werte. Die wichtigste Rolle nimmt hierbei die Kommunikation ein und ist damit untrennbar mit der Führungsrolle verwoben. Im Sinne einer umfassenden Analyse der Kommunikation geht es sowohl um verbale als auch um paraverbale und nonverbale Anteile. Nicht nur die von den Führungskräften benutzten Worte sollen als Vorbild dienen, sondern Mitarbeiter sollen auch über die Art des Sprechens genauso geführt werden. Dabei kommen der Intonation der Stimme und der Körpersprache eine besondere Bedeutung bei, weshalb Führungskräfte gegebenenfalls eine „face-to-face“-Kommunikation bevorzugen. Men (2014) stellte eine erhöhte Mitarbeiterzufriedenheit durch diese Art der Interaktion fest. Obgleich der Informationsaustauch beispielsweise via E‑Mail als effizient angesehen wird, bevorzugen nach White et al. (2010) auch Gruppen von Mitarbeitern die Kommunikation von Angesicht zu Angesicht.

Bedenken zur Kontrolle der Leistungserbringung und Stundenerfassung lassen sich unter fehlendem Vertrauen subsummieren. Obwohl Führungskräfte ihre Mitarbeiter weder ganztägig überwachen können (noch üblicherweise wollen), scheint es, dass der physische Anblick der Mitarbeiter an ihren Präsenzarbeitsplätzen zu einem stärkeren Gefühl der Kontrolle bei den Führungskräften beiträgt. Dem entgegengesetzt schwindet das Gefühl, den Überblick über Arbeitsstunden und Leistungserbringung zu behalten, sobald der Mitarbeiter an anderen Orten tätig ist. Dabei ist es unerheblich, ob dies im Homeoffice oder an ausgelagerten Standorten stattfindet. Die Beziehung zwischen Führungskraft und Mitarbeitern muss sich daher über gegenseitiges Vertrauen und nicht über reine top-down Kontrollmechanismen definieren wie es beispielsweise im „Modell zur Stärkung der Vertrauenskultur zwischen Führungskräften und ihren Mitarbeitern und Mitarbeiterinnen“ von Misamer und Thies (2017) ersichtlich ist.

Wie beschrieben, spielen die Komponenten Kommunikation und Vertrauen (im Sinne der Stundenerfassung und Leistungserbringung) eine zentrale Rolle. So unterschiedlich einzelne Unternehmen und ihre Rahmenbedingungen sind, so sehr ähneln sich diese als zentral erachteten Themenbereiche (Müller et al. 2017). Im Folgenden werden mögliche Lösungsstrategien dargelegt, wie sie im Sinne des Stands der Forschung im eigenen Unternehmen Anwendung finden können. Dabei streben die Autoren weder die Vollständigkeit aller verfügbaren Werkzeuge noch eine tiefere technische Analyse an. Die Beschreibung ist Grundlage für die empirische Studie, in deren Durchführung die potenzielle Anwendung und Kombination der Werkzeuge analysiert werden soll.

### Kommunikation

Zur Unterstützung der Kommunikation ist ein kostengünstiges, leicht in den Bestand integrierbares „Collaboration-Tool“ mit entsprechend hohen Sicherheitsstandards (insb. Multifaktorauthentifizierung) und den Funktionen Audio‑/Videotelefonie (Teambesprechungen/Konferenzen mit Screensharing) sowie Einzel- und Multichatfunktion Anforderung. Ein wichtiger Teil der Teamkultur ist die gemeinsame Ausrichtung der Aktivitäten, sowie die Abstimmung der künftig geplanten Umsetzungen. Hierzu gibt es regelmäßig wiederkehrende Teammeetings. Digital Leadership verlangt folglich eine adäquate Lösung zum Präsenztermin. Virtuelle Teambesprechungen müssen die Möglichkeit bieten, sein Gegenüber sowohl akustisch als auch bei Bedarf visuell verfolgen zu können.

### Vertrauen: Leistungserbringung, Stundenerfassung

In der Publikation von Bijlsma-Frankema und Costa (2005) fassen die beiden Autorinnen grundlegende Erkenntnisse in der Vertrauensforschung zusammen. Vertrauen kann der Kontrolle als Antagonist entgegengesetzt werden. Dabei wird implizit eine polare, umgekehrt proportionale Beziehung der beiden Parameter Vertrauen und Kontrolle verstanden. Patriarchale Führungsstile bedienten sich traditionell einer sichtbaren und umfassenden Kontrolle des Arbeitnehmers in Bezug auf Anwesenheit und Leistungserbringung. Die zweite Perspektive erlaubt die Annahme einer komplementären Beziehung der Parameter Vertrauen und Kontrolle. Es wird angenommen, dass klare Regeln und transparentes Monitoring bei der Bewertung von Eigen- und Fremdleistungen hilfreich sein können und dadurch zu einem gestärkten Vertrauensverhältnis führen. Kooperative Stile machen sich Kontrolle in Form von Monitoring des Outputs zunutze und fördern ein vertrauensvolles Arbeitsumfeld. Unter anderem definiert die Art der Tätigkeit, ob eine Verschiebung zu Gunsten des Vertrauens möglich ist. Der ohnehin stattfindende Wandel zu mehr Vertrauen in den einzelnen Mitarbeiter wird durch die Covid-19 Pandemie beschleunigt. Ein Arbeiten im reinen Vertrauenskontext, losgelöst von jeglicher Kontrolle, wird durch die rechtlichen Rahmenbedingungen – Arbeitszeitgesetze, Versicherungsschutz im Homeoffice, Haftungen – nicht umsetzbar sein. Dennoch müssen Führungskräfte den Wandel hin zum Vertrauen in Bezug auf die Arbeitsinhalte vollziehen.

In vielen Unternehmen wurden bereits vor der Diskussion um mögliche Arbeitsplätze „außerhalb“ des Hauptstandorts Aufträge in einem Dokumentationssystem erfasst. Ein Ticketsystem ist ein gängiges Modell in Unternehmen und sowohl Führungskräften wie auch Angestellten vertraut. Diesen Systemen gemein sind verschiedene Auswertungsmöglichkeiten, die Rückschluss über die Leistungserbringung erlauben, besonders dann, wenn die Systeme bereits über einen längeren Zeitraum die Aufträge, welche am Präsenzarbeitsplatz erfüllt wurden, erfasst. Kontrollmöglichkeiten über das Ticket-System sind beispielsweise die Anzahl offener Tickets pro Organisationseinheit/User und Durchschnittswerte der Rate für Tickets (Anzahl gelöster Tickets vs. Anzahl geöffneter Tickets) können verglichen werden. Dies kann entweder auf Abteilungen/Teams oder einzelne Personen heruntergebrochen werden. Damit sind Abweichungen erkennbar und können thematisiert bzw. behandelt werden. Eine transparente Auswertung kann dem einzelnen Mitarbeiter helfen, eine Selbstkontrolle durchzuführen und unterstützt damit das Vertrauen in Richtung der Führungskraft.

Rechtliche Rahmenbedingungen zur Unfallversicherung und des Arbeitszeitschutzes sind beispielsweise bei der Erfassung der Arbeitszeit dennoch die maßgebenden Parameter. Damit die Arbeitszeit entsprechend im Distance Working nicht manuell eingebucht werden muss, bedarf es einer adäquaten Lösung analog zur klassischen Stechuhr. In Unternehmen, wo eine automatische Erfassung der Arbeitszeit rechtlich zulässig ist, wird gerne ein System verwendet, bei welchem der Mitarbeiter die Buchungsart (Kommen, Gehen, Dienstgang, …) an der Stechuhr auswählt und die Auswahl im System durch Hinhalten des Mitarbeiterausweises einbucht. Dies kann für die Angestellten mit Distance Working auf Softwarebasis implementiert werden.

## Design und Evaluierung des Konzepts

Zur Entwicklung und Evaluation des Konzepts zur Steigerung der Akzeptanz von Distance Working bei Führungskräften wurde als Methode Design Science Research (DSR) verwendet. DSR, als vorherrschende konstruktionsorientierte Methode in der Wirtschaftsinformatik und Information System Disziplin, basiert in der gegenständlichen Anwendung im Wesentlichen auf Hevner et al. (2004) und Peffers et al. (2007). In Analogie zu den beiden Forschungszyklen (Rigor, Relevance) nach Hevner (2007) wurden im ersten Zyklus (Rigor) das Erkennen der Bedenken und Hindernisse der Führungskräfte in Bezug auf den Wandel der Führungsrolle im Distance Working sowie das Erkennen der nötigen Rahmenbedingungen in Form strukturierter Literaturrecherche zum Stand der Forschung durchgeführt. Hierfür wurden gängige wissenschaftliche Datenbanken wie beispielsweise AIS Digital Library (mit Fokus auf die Journale des „Senior Scholars’ Basket of Journals“), EBSCO Host oder ACM Portal und Metasuchmaschinen wie Google Scholar verwendet. Aufgrund der Aktualität konnte der überwiegende Teil der Publikationen den Jahren 2017 bis 2020 entnommen werden, ergänzt durch definitorische Literatur. Dabei wurden deutschsprachige und englische Titel in die Suche inkludiert. Zur Identifikation des Artefakts wurden 51 Artikel erfasst, 17 sind in das Design eingeflossen. Der zweite, praktische Zyklus (Relevance), die Beschreibung der technischen Umsetzung und die Beschreibung der Implementierung der erarbeiteten Lösungsstrategien, nimmt Bezug auf eine bespielhafte Umsetzung im betrieblichen Umfeld.

**Abb. 1:**
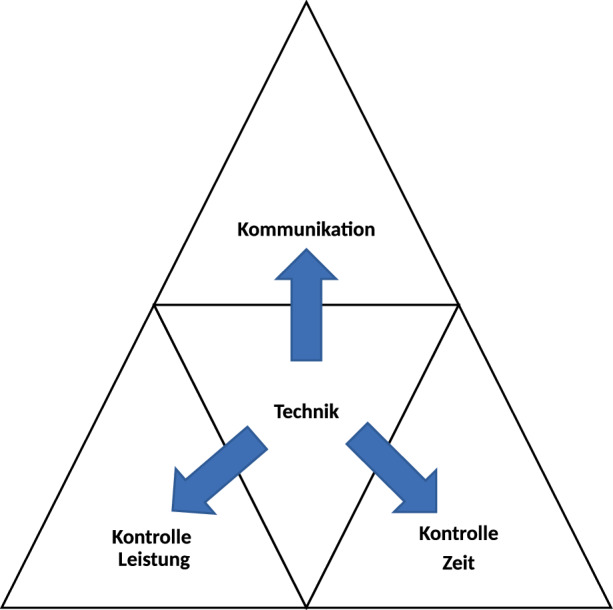
Artefakt vor der Evaluierung

Die Verknüpfung von Theorie und Praxis führte in einer ersten Designphase zu dem in Abb. 1 dargestellten Artefakt, bei welchem die drei zentralen Faktoren, die sich aus der Literaturrecherche ergeben haben und im Zuge der Designphase nachgeprüft werden konnten – Kommunikation, Kontrolle der Leistung und Kontrolle der Zeiterfassung – direkt von der Bereitstellung und dem reibungslosen Einsatz der Technik abhängen. Der Einsatz von technischen Lösungen ist die zentrale Komponente über die alle drei Faktoren gesteuert und reguliert werden. Daher ist davon auszugehen, dass ein optimaler Einsatz von technischen Lösungsmöglichkeiten Führungskräfte in der Akzeptanz des Digital Leadership bei Distance Working unterstützt.

Das Artefakt wurde im Rahmen von zehn Experteninterviews leitfadengestützt hinsichtlich dessen Relevanz bewertet, wobei es einer Tauglichkeitsprüfung entsprechend vorher festgelegter Evaluationskriterien nach Gregor und Hevner (2013) unterzogen wurde. Als Experten fungierten Führungskräfte aus den Branchen IT/Technik, Industrie und Soziale Arbeit, die jeweils mehrjährige Erfahrung in der Führung von Mitarbeitern am Präsenzarbeitsplatz hatten und sich pandemiebedingt vor die Herausforderung gestellt sahen, ihre Führungskompetenz hinsichtlich Distance Working zu erweitern. Als Ergebnis dieses Prozesses ist nun ein evaluiertes Artefakt geschaffen, auf welchem aufbauend die Akzeptanz einer Implementierung des Distance Working in Betrieben nach den Distance-Working-Phasen erhöht wird.

Die Evaluierung des Artefakts anhand der zehn Interviews hat in der qualitativen Inhaltsanalyse zu den folgenden sieben zentralen Kategorien geführt:

- Erfahrungen mit Distance Working
- Anforderungen an den Arbeitsplatz
- Unterstützung/Ablehnung vom Betrieb
- Vertrauen vs. Kontrolle
- Kommunikation
- Herausforderungen
- Zukunftsorientierung

In den beiden einleitenden Kategorien „Erfahrungen mit Distance Working“ und „Anforderungen an den Arbeitsplatz“ wurden grundlegende Erfahrungen und Einstellungen thematisiert. Es zeigte sich eine durchwegs positive Einstellung gegenüber Distance Working, unabhängig von den gemachten Erfahrungen und umfangreichem Wissen über die Notwendigkeit von Arbeitsplatzanforderungen zum Wohle der Mitarbeiter im Distance Working. In der Kategorie „Unterstützung/Ablehnung vom Betrieb“ zeigte sich klar, dass die Unterstützung von höchster Ebene unerlässlich für das Gelingen von Distance Working ist und Denkmuster, die den Mitarbeitern Produktivität außerhalb der Sichtweite von Führungskräften absprechen kontraproduktiv sind. Der Vorschlag von konkreten und evaluierbaren Pilotphasen in Betrieben wurde genannt und für umsetzbar erachtet. Eng mit dieser Kategorie verwoben präsentieren sich die Ergebnisse aus der Kategorie „Vertrauen vs. Kontrolle“, denn hier wurden die obig erwähnten Denkmuster in Frage gestellt. Der Begriff der Kontrolle muss zunächst abgegrenzt werden. Führungskräfte unterscheiden hier zwischen einer „Überwachung“ des Mitarbeiters und einer „Steuerung“ in dem Sinne, dass das Unternehmen vor jedweder Art Schaden zu bewahren ist. Darunter fallen zudem die Einhaltung gesetzlicher Auflagen (Bescheide), wie beispielsweise dem Arbeitszeitgesetz, und die Unterstützung von Mitarbeitern in der Priorisierung hinsichtlich wichtiger und dringlicher Tätigkeiten. Hierfür wurde der Begriff „Monitoring“ mit Hilfe geeigneter KPIs verwendet.

Grundsätzlich muss eine Vertrauensbasis zwischen Führungskraft und Mitarbeiter geschaffen sein, wobei Führungskräfte proaktiv zum Aufbau dieses Vertrauens beitragen und diesen Prozess laufend reflektieren müssen. Vertrauensbildende Maßnahmen bedürfen eine guten „Kommunikation“, welche in einer eigenen Kategorie erfasst wurde. In den Interviews zeigte sich klar, dass den Führungskräften eine virtuelle Meeting-Kultur nicht fremd ist und sie Informationsverteilung und Kommunikationsregeln in virtuellen Gesprächen mit Hilfe diverser Collaboration-Tools zu steuern wissen. Trotz der Tatsache, dass derartige Meetings als produktiv angesehen werden und durchaus Vorteile bieten, berichteten die Interviewpartner übereinstimmend, dass ihnen als Führungskraft der „Draht zu den Mitarbeitern“ abhandenkommt durch das Fehlen von außerdienstlichen Gesprächen, die in einem Arbeitsalltag an einem Präsenzplatz üblich sind. Führungskräfte, die einen modernen Stil pflegen, sehen gerade diese außerdienstlichen Gespräche nicht als produktionshemmend, sondern als vertrauensfördernd und in weiterer Folge produktionsfördernd an und sprechen sich daher für ein Dienstverhältnis aus, das sowohl Distance Working wie auch physische Präsenz an einem gemeinsamen Arbeitsort ermöglicht, um diese außerdienstlichen Gespräche weiterhin pflegen zu können.

Konkret nach „Herausforderungen“ in einer weiteren Kategorie befragt, nannten die Interviewpartner neben der Kommunikation die Tatsache, dass aufgrund der zugrundeliegenden Stellenbeschreibungen nicht jeder Arbeitsplatz für ein partielles Distance Working geeignet ist und durch die vermeintliche Ungleichbehandlung möglicherweise Unruhe im Team entstehen kann. Als eine weitere Herausforderung wurden oftmals unklare gesetzliche und versicherungsrechtliche Fragen genannt. In diesem Zusammenhang ist zu erwähnen, dass konkrete Regelungen hierzu offen und seit September 2020 in Diskussion seitens der Gesetzgeber sind. Konkret nach der „Zukunftsorientierung“, der letzten der sieben Kategorien, befragt, waren sich alle interviewten Führungskräfte darin einig, dass Distance Working eine zentrale Rolle in der weiteren betrieblichen Ausrichtung spielen wird, sei es, um Kosten zu minimieren durch den Wegfall von Raummieten, Gebäudekosten, Reisekosten oder Reisezeiten. Pandemiebedingt wird ein virtuelles Kundengespräch nicht als mangelnder Respekt interpretiert, sondern als Einhaltung eines hohen Sicherheitsstandards geschätzt. Qualifizierte Schlüsselkräfte jeder Generation lassen sich bei ihrer Entscheidung für oder gegen einen Arbeitsplatz immer öfter von dem firmenseitigen Angebot nach Distance Working beeinflussen.

## Ableitung von Erkenntnissen und Interpretation

Besonders erfreulich stellte sich der bereits spürbare durchwegs positive Tenor gegenüber Distance Working im Allgemeinen und gegenüber einem partiellen ortsungebundenen Arbeitsplatz im Speziellen dar. Die Führungskräfte konnten durch hohe Empathiefähigkeit die Vorteile für die einzelnen Mitarbeiter unterstreichen, behielten jedoch immer das gesamte Team und Teamgefüge im Blick. Einige konnten bereits die Untersuchung von Latniak (2017), welcher Vorteile in der ungezwungenen Anbindung von räumlich verteilten Teammitgliedern beschreibt, unterstützen. Neben den positiven Effekten für den einzelnen Mitarbeiter wurden auch jene für das Unternehmen positiven Auswirkungen, wie Kostenreduktion oder Erhalt der Wettbewerbsfähigkeit, erkannt und benannt wie bereits von Kötting (2019) erläutert.

Die Führungskräfte sehen, erkennen und benennen die Herausforderungen des Digital Leadership in ihren Kernpunkten der Kommunikation und im Vertrauen. Auffallend dabei ist, dass die virtuelle Kommunikation im Bereich der sachlichen Meetings geschätzt wird, bei der Sozialkommunikation aber auch für Führungskräfte als unzureichend eingestuft wird. Die interviewten Führungskräfte sind sich durchwegs der Wichtigkeit von außerdienstlichen Gesprächen bewusst und wissen die durch diese Form der Gespräche gewonnenen Informationen für ihre Aufgabe der Führung gut zu nutzen. Bei einem partiellen Distance Working besteht die Möglichkeit der face-to-face Kommunikation für außerdienstliche Gespräche weiterhin und genau aus diesem Grund sprechen sich alle Interviewpartner für diese Variante – partiell statt gänzlich ortsungebundenen Dienstverhältnisses – aus und sehen sich ihrer Führungsrolle weiterhin gewachsen.

Im Punkt Vertrauen, einer weiteren Herausforderung, die Führungskräfte in Bezug auf das Digital Leadership benennen, gibt es eindeutig ein Spannungsfeld im Bereich Leistung und Stundenerbringung, wobei hier die Spannung mehr mit den gesetzlichen Rahmenbedingungen zu Tage tritt als Spannungen mit den Mitarbeitern. Die momentanen Arbeitszeitgesetze lassen nur begrenzten Spielraum zu, weswegen die Führungskräfte zwar einem ortsungebundenen Dienstverhältnis zustimmen, eine zeitungebundene Variante jedoch nicht befürworten können. Viele Interviewpartner konnten jedoch die Theorie zur Stärkung der Vertrauenskultur zwischen Führungskräften und ihren Mitarbeitern von Misamer und Thies (2017) bestätigen und sahen sich keineswegs einem Kontrollverlust ausgesetzt. Ein Monitoring im Sinne des Unternehmens wird als Aufgabe aktiv wahrgenommen und ausgeführt, dem Mitarbeiter auf individueller Ebene aber das nötige Vertrauen entgegengebracht und vorgelebt.

Auch Führungskräfte müssen in ihre Rolle eines Digital Leader hineinwachsen. Je klarer die Rahmenbedingungen hierfür sind, umso leichter fällt es ihnen. Gerade im Bereich der IT spielt für die Führungskräfte selbst der verstärkte Einsatz von Collaboration-Tools keine tragende Rolle in der Entscheidung für oder gegen ein partiell ortsunabhängiges Dienstverhältnis, sei es für Kommunikationszwecke oder für Monitoringaufgaben. Der Einsatz der Technik wird als grundlegender Baustein vorausgesetzt. Viel wichtiger werden klare Regeln im Umgang mit Kommunikation und Information gesehen, sowie rechtliche und/oder betriebliche Vereinbarungen, die Sicherheit schaffen und Bedenken abbauen.

Die interviewten Führungskräfte sind sich der Unsicherheiten auf Seiten der Mitarbeiter bewusst und sind bemüht, diese im Rahmen von Schulungen und klaren Vorgaben fit für ein partielles Distance Working zu machen. Gleichzeitig sind sie sich ihrer Verantwortung bewusst, diejenigen Mitarbeiter zu schützen, die dem hohen Erwartungsdruck an Eigenverantwortlichkeit, Organisationsgeschick und selbstbestimmtem Zeitmanagement nicht standhalten würden. Auch partielles Distance Working darf nur in einem ausgewogenen Verhältnis ein Angebot, aber niemals einen Zwang darstellen, so, wie es bei den ersten Maßnahmen zur Eindämmung der Epidemie, im D‑A-CH Raum, beginnend in Österreich ab 15. März 2020, größtenteils gehandhabt wurde. Durch die aktuellen Regelungen zum Homeoffice wird diese Freiwilligkeit deutlich gestärkt.

## Fazit und Ausblick

**Abb. 2:**
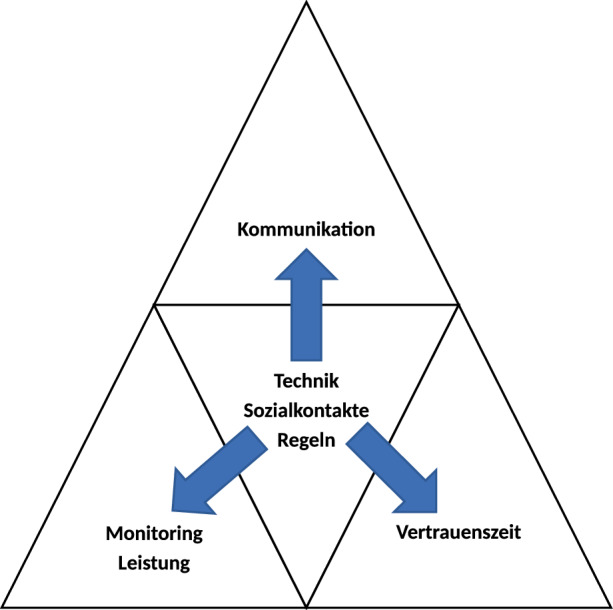
Artefakt nach Evaluierungsphase

Entgegen der ursprünglichen Annahme im Design des Artefakts vor der Evaluierungsphase, dass den technischen Lösungen (z. B. Collaboration-Tools) eine tragende Rolle beigemessen wird in der Erhöhung der Akzeptanz von partiell ortsunabhängigen Dienstverhältnissen, stellt sich in der Zusammenschau der Ergebnisse heraus, dass klar kommunizierte Regeln von Seiten des Gesetzgebers und von Seiten des Betriebes sowie die Möglichkeit der Aufrechterhaltung der physischen Sozialkommunikation ebenfalls dominierende Faktoren sind. Die Rolle des Digital Leaders wird umfassender definiert und gelebt als im Sinne eines Zeit- und Leistungskontrolleurs, weswegen etwa Bedenken wie Kontrollverlust auf Seiten der Führungskräfte oder mangelnde Leistungserbringung auf Seiten der Mitarbeiter keine Rolle spielen. Die bloße Anwendung von ausgereifter Technik zur Steuerung der Faktoren Kommunikation, Leistung und Zeiterfassung wird dem Bild eines Digital Leader nicht gerecht. Der moderne Digital Leader möchte als Partner wahrgenommen werden, der den direkten sozialen Kontakt sucht und nicht scheut, gleichzeitig aber die Einhaltung der Regeln überwacht und Leistung im Sinne eines Monitorings überwacht (Abb. 2). Wird den Führungskräften zugestanden, innerhalb klar und transparent kommunizierter gesetzlicher und betrieblicher Regeln mit ihren Mitarbeitern vertrauensvoll zu kooperieren, erhöht sich die Akzeptanz bei Führungskräften in Bezug auf ihre Rolle des Digital Leadership bei einer Mitarbeiterführung von partiell ortsunabhängigen Dienstverhältnissen.

## Footnotes

1 Distance Working wird in dem gegenständlichen Beitrag als Begriff für partiell orts- und zeitunabhängiges Arbeiten eingeführt, wobei aufgrund diverser arbeitsrechtlicher Regelungen im D‑A-CH Raum eine völlig freie Arbeitszeitverlagerung auf Nacht- oder Wochenendstunden ausgeschlossen ist und eine gewisse Erreichbarkeit zu definierten Kernstunden gewährleistet sein muss.
